# Down-regulation of C12orf59 is associated with a poor prognosis and VHL mutations in renal cell carcinoma

**DOI:** 10.18632/oncotarget.6829

**Published:** 2016-01-07

**Authors:** Jun Xie, Chuangzhi Zhu, Jianting Wu, Cailing Li, Liya Luo, Lingling Xia, Xianxin Li, Yaoting Gui, Zhiming Cai, Zesong Li

**Affiliations:** ^1^ Shenzhen Key Laboratory of Genitourinary Tumor, Shenzhen Second People's Hospital, First Affiliated Hospital of Shenzhen University, Shenzhen 518035, China; ^2^ Peking University Shenzhen Hospital, Shenzhen 518036, China

**Keywords:** renal cell cancer (RCC), C12orf59, von Hippel-Lindau (VHL), prognosis, survival

## Abstract

C12orf59 is newly identified gene in kidney. However, the relation of C12orf59 expression and clinic features is unknown. Here, our study showed that *C12orf59* was broadly expressed in normal human tissues with high expression levels in kidney while its expression is beyond detectable in a panel of cancer cell lines. C12orf59 expression in RCC was significantly decreased compared with corresponding adjacent noncancerous tissues (*P* < 0.01). The decreased C12orf59 expression was correlated with lymph node status (*P* < 0.05), distant metastases (*P* < 0.05), poor survival (*P* < 0.001) (HR 3.00; 95% CI, 1.29–7.53), VHL non-sense mutations or frame-shift mutations (*P* < 0.01), and UMPP gene non-sense mutations or frame-shift mutations (*P* = 0.01). Thus, we propose that the decreased C12orf59 expression status is a prognostic biomarker of ccRCC and cooperates with the loss of VHL all the while promoting renal carcinogenesis.

## INTRODUCTION

Renal cell carcinoma (RCC), the third most common malignancy of the genitourinary system, accounts for 3% of all adult malignancies [1]. The incidence of RCC is increasing in most areas of the world for which statistics are available for recent decades [2, 3]. Localized RCC is potentially curable with surgical resection of the diseased tissue, but 30% of patients develop metastatic disease after surgery. Metastatic RCC remains largely incurable due to its resistance to chemotherapy and radiation [4]. The median survival rate of metastatic RCC patients is 1.5 years, and the five-year survival rate is less than 10% [5].

Clear cell renal cell carcinoma (ccRCC) is the most common subtype of RCC, accounting for 60 to 80% of all RCCs [6]. The loss of function of the von Hippel–Lindau (*VHL*) tumor suppressor gene characterizes ccRCC [7–9]. VHL is a multifunctional protein that acts as an adaptor for different molecular and subcellular complexes. The best-characterized function of VHL is its role as the substrate recognition component of the E3 ubiquitin protein ligase complex that targets the α-subunit of the hypoxia-inducible factor (HIF) for proteolytic degradation destruction [10]. In ccRCC, the loss of VHL leads to up-regulation of HIF-α–mediated transcriptional programs that favor metastatic processes [11].

Recent studies identified several new ccRCC genes, including *UTX*, *JARID1C*, *SETD2*, and *BAP1* [12–16]. These studies have heralded a marked expansion in our understanding of the genetic landscape of ccRCC. However, the underlying molecular mechanisms of renal carcinogenesis remain unclear. It is still necessary to identify new signature genes and specific biomarkers in order to provide potential targets for RCC early detection. Furthermore, these targets will allow surveillance of tumor progression, and prediction of patient prognoses [17–21].

The *C12orf59* gene (Chromosome 12 open reading frame 59, also termed *FLJ31166* [22] or *MGC111385* [23]), localized on Chromosome 12p13.2, was first cloned in 2002 [23]. The open reading frame spans 12,847 bp, consists of 5 exons, and has 7 transcripts. Human *C12orf59* mRNA was associated with the RNA-binding protein HuR [24] and was predicted to encode transmembrane proteins [26]. The classical *C12orf59* mRNA (NM_153022.2) is 2800 bp long and encodes a 163 amino acid protein with a calculated relative molecular mass of approximately 18 kDa (NP_694567.1) [26]. Recently study indicated that the expression of *C12orf59* was decreased in RCC [27]. However, the expression and physiological roles of C12orf59 have not been investigated to date.

Here, we report that C12orf59 is frequently decreased at the mRNA and protein levels in a panel of genitourinary cancer cell lines and ccRCC. We found that the decreased C12orf59 expression is associated with renal cell carcinoma clinico-pathological parameters. Therefore, C12orf59 protein level change might be used for clinic marker for RCC early detection, surveillance of tumor progression, and prediction of patient prognoses.

## RESULTS

### Tissue distribution of C12orf59 and localization of C12orf59

**Figure 1:**
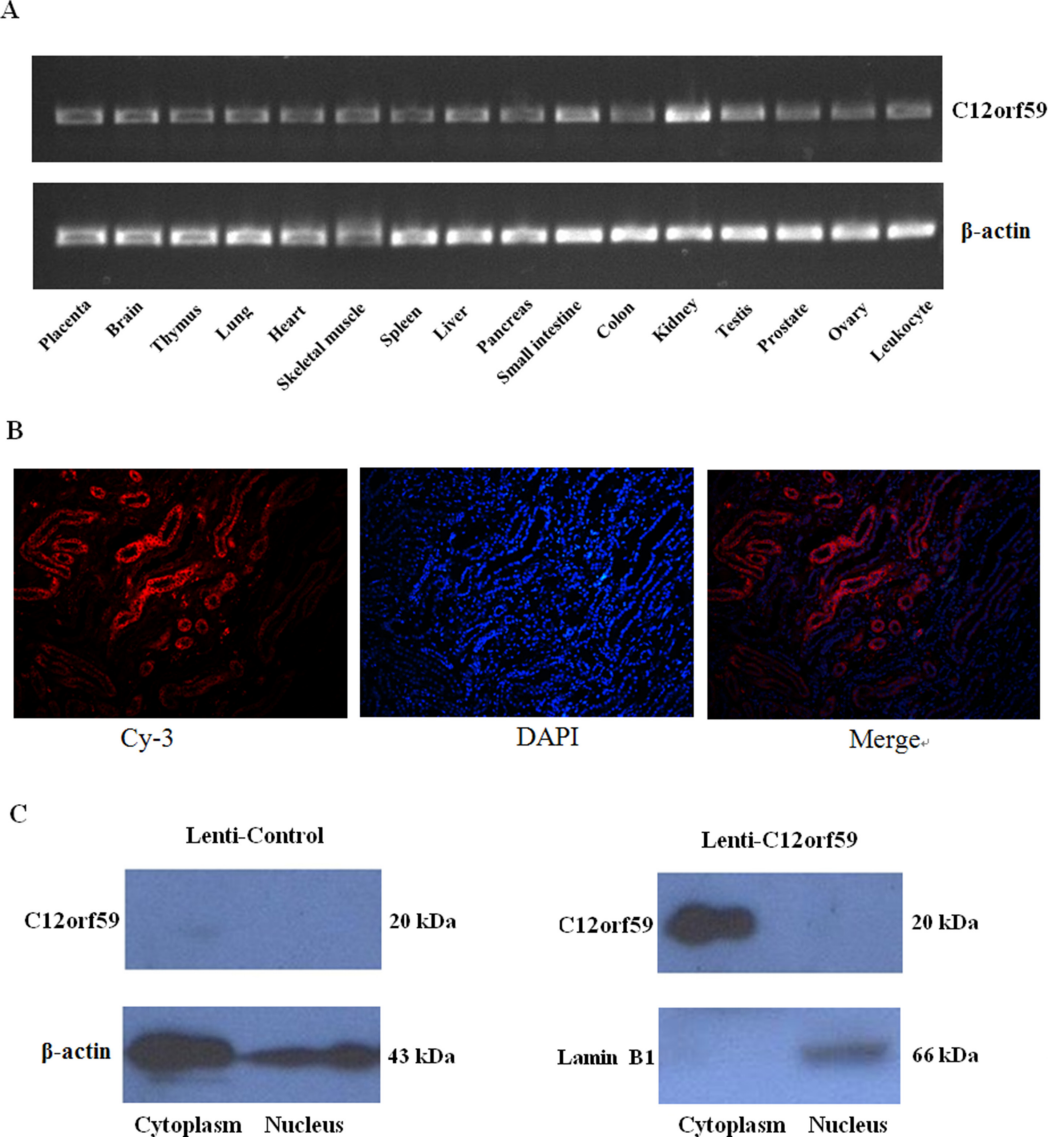
Tissue distribution of *C12orf59* mRNA expression and subcellular localization of the C12orf59 protein in kidney tissues (**A**) The *C12orf59* mRNA levels were analyzed in a panel of human adult tissues using RT-PCR. *β-actin* was used as a loading control. (**B**) Immunofluorescence staining captured by fluorescence microscope, including Cy-3, DAPI, and merged images. The C12orf59 protein was observed mainly in the cytoplasm. (**C**) Cytoplasmic and nuclear extracts were obtained from ACHN cells transfected with a lentiviral vector-encoding *C12orf59* for western blot analysis, and the C12orf59 protein was detected primarily in the cytoplasm fractions, with no detectable C12orf59 protein in the nuclear fractions.

C12orf59 is newly identified gene. However, its tissue distribution and subcellular location remains unclear. Our results showed that *C12orf59* mRNA was broadly expressed in the majority of the human normal tissues detected, but it was most highly expressed in the kidney (Figure 1A). Immunofluorescence staining showed that C12orf59 was located in the cytoplasm (Figure 1B). Furthermore, cytoplasmic and nuclear extracts were obtained from renal cancer cells stable transfected C12orf59 expression lentivirus. The western blot results showed that the C12orf59 protein was primarily detected in the cytoplasm fractions, with no detectable C12orf59 protein in the nuclear fractions (Figure 1C).

### Loss of C12orf59 expression in the RCC cell lines and ccRCC

**Figure 2:**
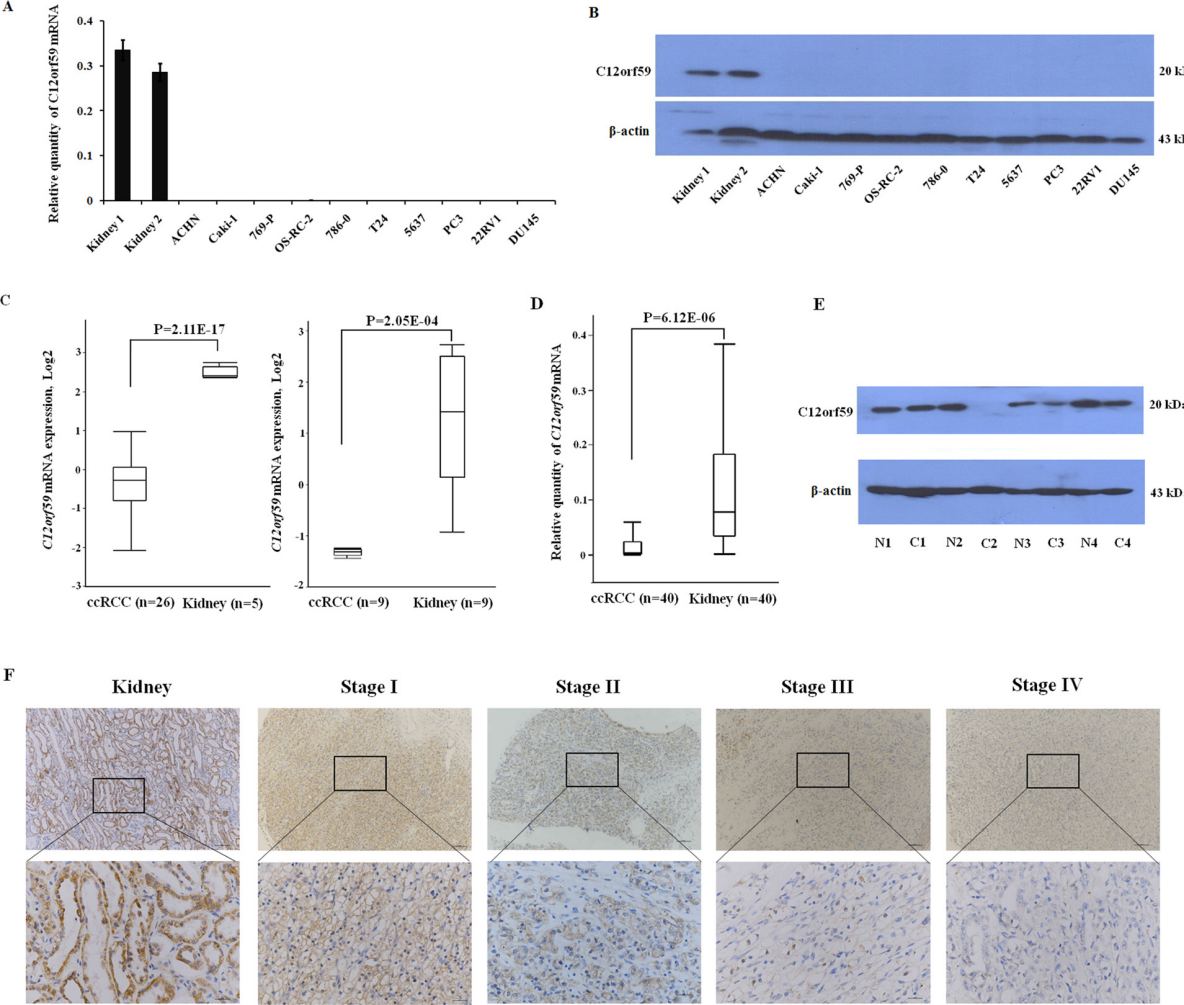
C12orf59 is down-regulated in ccRCC (**A**) The quantitative assessment of *C12orf59* mRNA levels by real-time PCR in normal kidney tissues, renal cancer cell lines (ACHN, Caki-1, 769-P, OS-RC-2, 786-0), bladder cancer cell lines (T24, 5637), prostate cancer cell lines (PC3, LNCaP, DU145). The data are shown as the mean ± S.D. for three independent quantifications. (**B**) The C12orf59 protein in these genitourinary cancer cell lines was detected by western blot. (**C**) Oncomine was used to analyze the microarray data previously published by Yusenko [28] (left) and Lenburg [29] (right) using standard settings. (**D**) The expression of *C12orf59* in 40 ccRCC and paired noncancerous tissue samples are determined by real-time RT-PCR (*P* < 0.0001). (**E**) Representative results for the C12orf59 protein in ccRCC and paired noncancerous tissue samples by western blot. (**F**) Representative immunohistochemical staining for C12orf59 expression in patient normal and ccRCC tissue stage I to IV.

To test the expression status of *C12orf59* in the cancer cell lines, we performed a RT-PCR analysis on a panel of genitourinary cancer cell lines (five renal cancer cell lines: ACHN, Caki-1, 769-P, OS-RC-2 and 786-0; two bladder cancer cell lines: T24 and 5637; three prostate cancer cell lines: PC3, LNCaP and DU145). Our results demonstrated that no detectable *C12orf59* mRNA was observed in these cancer cell lines (Figure 2A). This result was confirmed by western blot analysis (Figure 2B).

We previously analyzed the expression profile of ccRCC using deep sequencing technology and revealed that *C12orf59* was decreased in a majority of the ccRCC samples compared to the paired non-tumor tissues [27]. To investigate whether *C12orf59* expression is altered during carcinogenesis, we performed an *in silico* analysis of *C12orf59* expression in human normal kidney tissues and ccRCC using microarray expression studies published in Oncomine(31). Two independent studies showed that the *C12orf59* expression was significantly decreased in the ccRCC samples compared with the normal tissues (*p* < 0.01) (Figure 2C) [28, 29]. We analyzed the expression of *C12orf59* in additional 40 paired ccRCC samples and non-tumor tissues. The results showed that the *C12orf59* mRNA levels were silenced or strongly decreased in 33 of the 40 ccRCC samples with an overall 4.5-fold decrease in ccRCC compared to the paired non-tumor tissues (*P* < 0.001) (Figure 2D). The results were confirmed by western blotting (Figure 2E). IHC staining for C12orf59 protein expression in matched tumor and normal tissue confirmed the results and decreased expression in tumor samples across all stages (Figure 2F).

### Loss of C12orf59 is correlated with tumor stage, metastasis and poor prognosis

We examined the possible correlations between the expression levels of C12orf59 and the clinical features of ccRCC in 208 primary ccRCC samples. As summarized in Table 1, the patients were assigned to two subgroups according to the expression levels of C12orf59: the low expression group (*n* = 114) and the high expression group (*n* = 94). The C12orf59 low expression group showed more frequent regional lymph node metastases (*P* = 0.04), distant metastases (*P* = 0.016) and late tumor stage (*P* = 0.014) development than the high expression group. The Spearman correlation analysis (Supplementary Table S2) showed that the C12orf59 expression level was significantly inversely correlated with regional lymph node metastases (*P* = 0.14, *P* = 0.04), distant metastases (*P* = 0.17, *P* = 0.02), and late tumor stage (*P* = 0.17, *P* = 0.01). No significant correlations between the expression levels of C12orf59 with age, gender and tumor size were observed (Table 1, Supplementary Table S2).

**Table 1:** Correlation between C12orf59 expression and the clinico-pathologic features of patients with clear cell renal cell carcinoma

Clinico-pathologic variables	No. of cases	C12orf59 expression		*χ*^2^	*p*
		low	high		
All cases	208	114	94		
**Gender**					
Male	138	81	57	2.503	0.114
Female	70	33	37		
**Age**					
> 50	120	71	49	2.176	0.140
< 50	88	43	45		
**Size**					
> 7 cm	49	24	25	0.879	0.348
< 7 cm	159	90	69		
**Primary tumor stage**					
T1–2	171	87	84	5.996	0.014
T3–4	37	27	10		
**Lymph node status**					
N0	190	100	90	4.198	0.040
N+	18	14	4		
**Distant metastasis**					
M0	194	102	92	5.789	0.016
M1	14	12	2		

**Figure 3:**
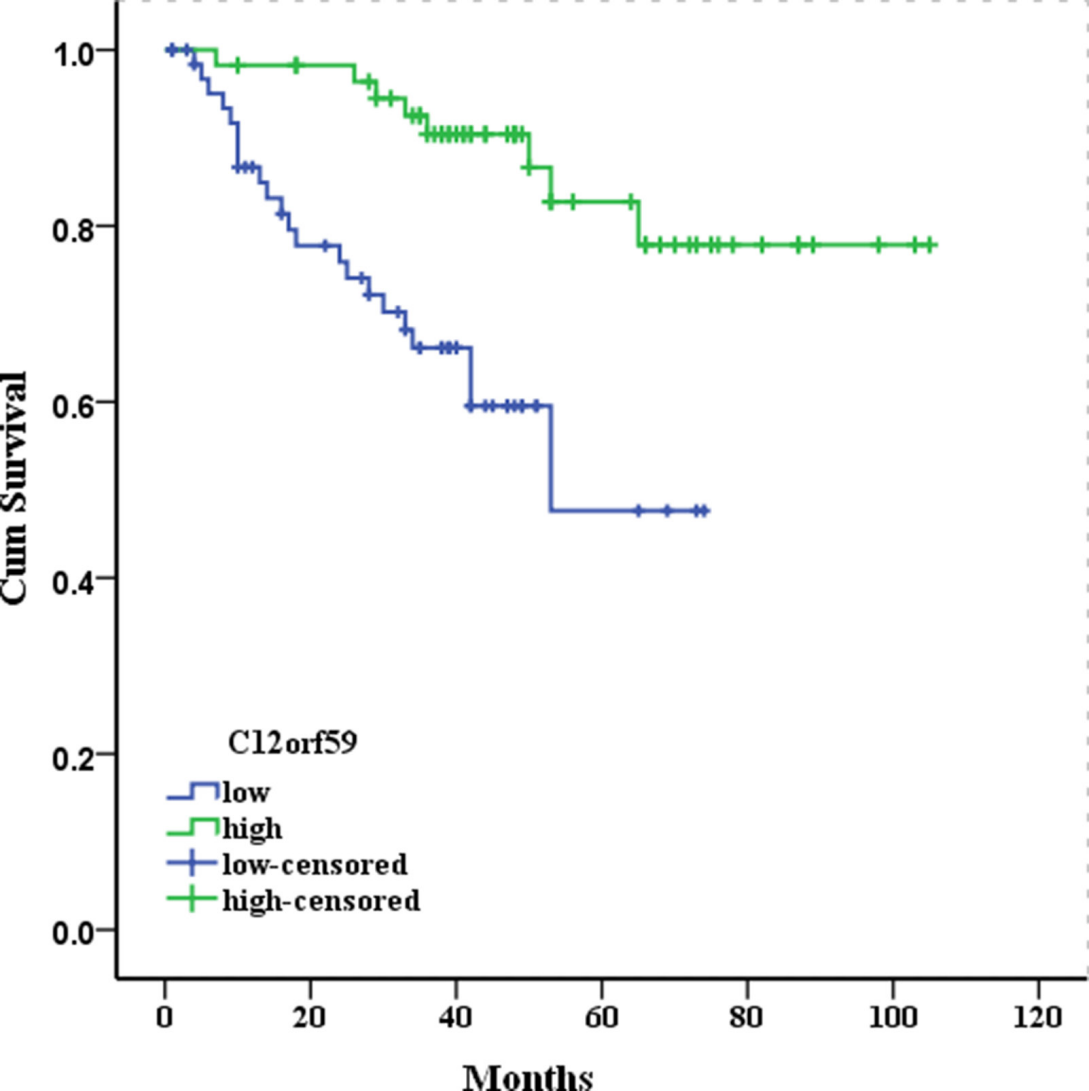
Kaplan-Meier survival curves for 122 patients with clear cell renal cell carcinoma according to C12orf59 expression The cumulative 5-year survival rate was 78.9% in the high protein expression group (*n* = 57) and 52.5% in the low C12orf59 expression group (*n* = 65) (*P* < 0.001).

We examined the correlation between the expression status of C12orf59 and the ccRCC prognosis using 122 of the 208 primary ccRCC samples with available follow-up data. As shown in the overall survival curve, the patients in the C12orf59 low expression group (five year survival rate, 52.5%) had a significantly poorer prognosis than those in the C12orf59 high expression group (78.9%, *P* < 0.001; Figure 3). The median survival time for the patients in the C12orf59 low expression group was 50.1 months as compared to 91.4 months for those in the C12orf59 high expression group.

The univariate analysis of the overall survival revealed that the relative level of C12orf59 expression, primary tumor stage, the regional lymph node metastases and distant metastases were prognostic predictors (Table 2). The variables with a *P* value < 0.05 were selected for the multivariate analysis. The multivariate analysis showed that the relative level of C12orf59 expression, the primary tumor stage, and distant metastases were independent prognostic predictors. The patients with high C12orf59 protein levels in the tumors had a better prognosis than the patients with low C12orf59 protein levels (RR: −1.40, 95% CI: 0.11–0.57, *P* = 0.009; Table 2).

**Table 2:** Univariate and multivariate analyses for overall survival

Clinico-pathologic variable	Univariate analysis			Multivariate analysis		
	RR	95.0% CI	*P* value	RR	95.0% CI	*P* value
Gender (male/female)	0.57	0.72–4.31	0.216	–	–	–
Age (>/< 50 years)	−0.002	0.49–2.04	0.995	–	–	**–**
Size (>/< 7 cm)	−0.52	0.33–1.07	0.082	–	–	–
Primary tumor stage (T1–2/T3–4)	1.37	1.92–8.10	< 0.001***	0.94	1.11–5.92	0.028*
Lymph node status (negative/positive)	1.49	2.0–9.92	< 0.001***	0.59	0.71–4.54	0.231
Distant Metastasis (negative/positive)	1.59	2.06–11.61	< 0.001***	1.31	1.48–9.28	0.005**
C12orf59 (low/high)	−1.40	0.11–0.57	0.001**	−1.18	0.13–0.74	0.009**

Abbreviations: RR; Relative risk, CI; Confidence interval, *P < 0.05, **P < 0.01, ***P < 0.001

### Genetic deletion or mutation of *C12orf59* is not detected in renal cancer cell lines or primary renal cancer tissues

To determine whether genetic alterations contribute to the silencing of *C12orf59*, we performed sequence screening on the exons and exon-intron junctions of *C12orf59* in 5 renal cancer cell lines and 100 paired ccRCC samples using PCR and direct sequencing. The results showed no somatic mutations/deletions of *C12orf59* in the five human renal cancer cells and the 100 paired ccRCC samples, suggesting that the decreased C12orf59 expression might not be caused by genetic alterations.

### C12orf59 expression is correlated with mutations of the genes encoding the ubiquitin-mediated proteolysis pathway (UMPP)

**Figure 4:**
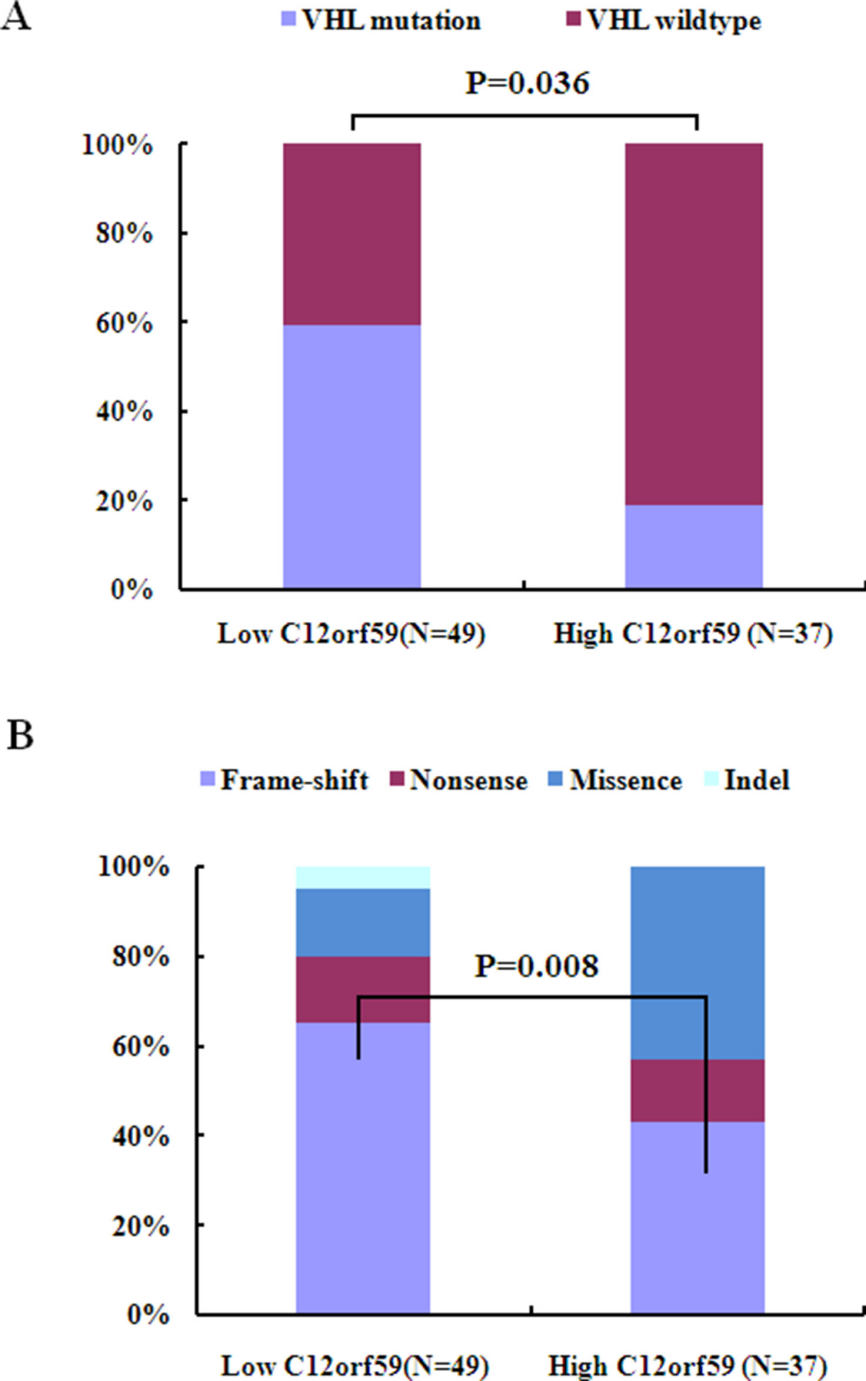
C12orf59 expression is associated with *VHL* mutation in 88 ccRCC samples (**A**) The *VHL* mutation frequency in the C12orf59 low expression group and the C12orf59 high expression group. (**B**) The distribution of *VHL* mutation types in the C12orf59 low expression group and the C12orf59 high expression group.

As the mutation status of exons was known for 86 ccRCCs [30], we investigated whether C12orf59 expression is correlated with the mutation status of the genes encoding the ubiquitin-mediated proteolysis pathway (Supplementary Table S3). We determined that the fraction of UMPP mutated tumors in the C12orf59 low expression group (30/49, 61%) was similar to that in the C12orf59 high expression group (15/37, 41%) (Supplementary Table S3), but the fraction of frame-shift mutations and non-sense mutations were more frequent in the C12orf59 low expression group than that in the C12orf59 high expression group (39% *vs.* 11%, *p* = 0.006). The fraction of the *VHL* mutated tumors, including whole mutations or frame-shift mutations and non-sense mutations, in the C12orf59 low expression group were more frequent than in the C12orf59 high expression group (41% *vs.* 19%, *p* = 0.036; 33% *vs.* 8%, *p* = 0.008) (Figure 4 and Supplementary Table S4). These results indicate that there is a significant correlation between the lack of C12orf59 expression and the *VHL* and UMPP mutations.

### C12orf59 expression is not correlated with mutations of the chromatin remodeling genes

Due to recent sequencing efforts have identified frequent mutations of chromatin remodeling and histone-modifying genes in ccRCC [12, 14, 30], we analyzed whether C12orf59 expression is associated with the mutation status of these genes (Supplementary Table S5). The results showed that the mutation fraction of the chromatin remodeling genes in the C12orf59 low expression group (22/49, 44.9%) and in the C12orf59 high expression group in ccRCC (18/37, 48.6%) was similar, and the fraction of frame-shift mutations and non-sense mutations were similar (34.7% vs. 35.1%). These data indicate that the lack of C12orf59 expression is not correlated with mutations of the chromatin remodeling genes.

### C12orf59 expression is not correlated with HIF1α and HIF2α in ccRCC

Because the HIF1α and HIF2α protein levels were known for the 86 ccRCC samples [30], we evaluated the potential correlations between the protein expression patterns of C12orf59, HIF1α and HIF2α in ccRCC. As shown in Figure S2, 59.4% (22/37) of the tumors in the C12orf59 high expression group were HIF1α positive, and 26.5% (13/49) of the tumors in the low expression group were HIF1α negative (Supplementary Figure S2). The correlation between the expression of C12orf59 and HIF1α was not significant (*P* = 0.24). In addition, 78.4% (29/37) of the tumors in the C12orf59 high expression group were positive for HIF2α, and 32.6% (16/49) of the tumors in the C12orf59 low expression group were HIF2α negative (Supplementary Figure S2). The correlation between the expression of C12orf59 and HIF2α was not significant (*P* = 0.33). Thus, there was no correlation between the expression of C12orf59 and that of HIF1α and HIF2α.

## DISCUSSION

We found that the *C12orf59* gene was expressed in almost all the tissues, with the highest levels of expression found in the kidney, and that C12orf59 is predominantly a cytosolic protein (Figure 1B and 1C). The C12orf59 protein is highly conserved among humans, chimpanzees, cows, pigs, mice and rats (Supplementary Figure S1). This ubiquitous expression and conserved sequences of C12orf59 suggested that it might have an important role in the regulation of cell progression.

Our previous study showed that *C12orf59* was decreased in a small cohort of ccRCC samples [27]. In the present study, we found that the expression of C12orf59 was lacking in a panel of genitourinary cancer cell lines (Figure 2A and 2B). By using the *silico* gene expression data screening Oncomine database [31], we confirmed that the *C12orf59* expression in ccRCC is decreased compared with normal tissues (Figure 2C). The results were validated by analyzing the expression of *C12orf59* in 40 ccRCC samples and paired noncancerous tissues (Figure 2D and 2E).

To investigate whether C12orf59 expression is correlated with the progression of ccRCC, the C12orf59 expression levels and the clinical pathological characteristics of 208 patients with ccRCC were compared by immunohistochemistry. The results showed that the decreased *C12orf59* expression is significantly correlated with the primary tumor stage, lymphatic invasion (*R* = 0.14, *P* = 0.04), and distant metastases (*R* = 0.17, *P* = 0.02) (Table 1), suggesting that decreased C12orf59 expression might be important for the acquirement of malignancy potential in ccRCC. The multivariate analysis revealed that the decreased *C12orf59* expression was a worse independent prognostic factor in ccRCC patients (Figure 3 and Table 2). To our knowledge, this report is the first to demonstrate that *C12orf59* has prognostic value as an immunohistochemical biomarker of patient survival in human cancer.

The genetic mechanism of the decreased expression of C12orf59 was investigated by direct sequencing of the gene promoter and exons. No somatic mutations/deletions of *C12orf59* in the five human renal cancer cell lines and 100-paired ccRCC samples were identified. Whether molecular mechanisms other than mutations, such as promoter hypermethylation, might contribute to C12orf59 silencing remains unknown, but the possibility might reflect genetic and epigenetic events and needs further exploration.

Our analysis revealed that low C12orf59 expression is significantly correlated with the mutation status of genes encoding the ubiquitin-mediated proteolysis pathway, especially with the *VHL* mutation, but not with mutations of the chromatin remodeling genes. C12orf59 expression is not correlated with HIF1α or HIF2α, two crucial hypoxia regulatory factors, which were the most intensively investigated VHL target genes. These data suggest that C12orf59 expression is not inhibited by the canonical hypoxia pathway. This raised the possibility that the decrease of C12orf59 expression may be provoked by the loss of VHL in a HIF-independent fashion by the same mechanism that VHL influence the p53 expression [32]. We speculated that, in some instances, the decreased *C12orf59* expression and the loss of VHL might cooperate to promote the development of ccRCC because VHL inactivation alone is insufficient for tumor initiation [33, 34].

In a word, our data provides the first evidence that the loss of *C12orf59* expression is a common feature of ccRCC that is correlated with increased aggressive tumor behavior and predicts poor survival outcomes of patients. Decreased C12orf59 expression and the loss of VHL may cooperate to promote renal carcinogenesis.

## MATERIALS AND METHODS

### Cell culture

The human renal cell carcinoma cell lines (786-O, OS-RC-2, ACHN, 769-P, and Caki-1), bladder cancer cell lines (T24, 5637), and prostate cancer cell lines (PC3, LNCaP, DU145) were purchased from the American Type Culture Collection (ATCC). All the cell lines were cultured in accordance with the supplier's instructions.

### Construction of lentivirus vector and lentivirus infection

*C12orf59* cDNA was cloned into pGV lentivirus vector (Genechem Incorporation, Shanghai, China). The resulting lentivirus vector together with pHelper 1.0 and pHelper 2 vectors were cotransfected into 293FT cells to generate lentiviral stock, and pGV empty vector served as negative control. Forty-eight hours after transfection, supernatant harboring lentiviruses were collected. ACHN cells were infected by lentiviruses in 6-well plates by applying infection cocktail. After 48 hours, infected cells were selected to expand culture for further investigation by using 2 μg/ml of puromycin. Expression of C12orf59 was verified by using real-time PCR and Western blotting.

### Tissue samples

Tissue samples from the tumors with matched normal controls were surgically resected at member institutions of the Urinogenital Cancer Genomics Consortium (UCGC) in China as described previously [30]. Of 100 ccRCC and paired normal kidney tissues, only 86 paires with detailed information were selected for further analysis [30]. An additional 122 paraffin-embedded RCC tissue samples with follow-up data were collected from patients between 1999 through 2007. Each patient had provided written informed consent prior to study participation. This study protocol was carried out with ratification by the Ethics Committee of Shenzhen Second People's Hospital. Of these 208 patients, 138 were men, and 70 were women. The median age of the patients was 53 years (range, 4–83 years). The median follow-up time was 39 months (range, 1–105 months). Information regarding the gender, age, stage of disease, and histopathological factors was obtained from the medical records. All the tumors were confirmed as ccRCC by the clinical pathology department of the hospital, and the cases were staged according to the tumor node metastasis staging system.

### RT-PCR

The levels of *C12orf59* mRNA in different human adult tissues (Human Multiple Tissue cDNA Panels, I and II) were determined by semi-quantitative PCR with *C12orf59* primers and *β-actin* primers in accordance with the manufacturer's instructions. The levels of *C12orf59* mRNA in the cancer cell lines and the tumor and paired normal tissues were determined by a SYBR Green-based real-time PCR assay with *C12orf59* primers, and *β-actin* was used as the internal control. The PCR mixture was initially incubated at 94°C for 2 minutes, followed by 40 cycles of denaturation at 94°C for 15 seconds and annealing and extension at 68°C for 30 seconds. The assay was carried out three times in triplicate using the following primer sets: *C12orf59*-F: 5′-CAGCACTCTCCAGAGCACTATCA-3′ and *C12orf59*-R: 5′-TGGCTACT GTGAAGCGACTCAT-3′; *β-actin*-F: 5′-GGCACCACACCTTCTACAATGAG-3′; and *β-actin*-R: 5′-GGATAGCACAGCCTGGATAGCA-3′. The relative expression level of *C12orf59* was determined using the 2^−ΔΔCt^ method [35].

### Database mining

The Oncomine database [31] was used for retrieving the alterations in the mRNA expression levels in cancers and corresponding disease-free normal and/or normal adjacent tissues.

### Immunofluoescence

For immunofluorescence, the sections were deparaffinized in xylene and rehydrated through graded alcohols, then boiled in 10 mM citrate buffer (pH 6.0) for 30 min for the antigen retrieval. The endogenous hydrogen peroxidase was blocked by treating the slides with 3% hydrogen peroxide and incubating them for 20 min. The section were washed three times in PBS and incubated in TBS containing 5% BSA for 30 min at room temperature. The section were incubated with a 1:200 dilution of rabbit polyclonal anti-C12orf59 (ABGENT, CA) in 0.5% BSA in PBS overnight at 4°C. The section were washed three times with cold PBS and incubated with a 1:1000 dilution of the secondary antibody donkey anti-rabbit IgG conjugated to Cy3 (EarthOx, USA) in 0.5% BSA in PBS for 1 h at room temperature. After the section were washed three times with PBS and once with water, the nuclei were stained with 4′,6-diamidino-2-phenylindol (DAPI) (Sigma, USA). Immunofluorescence was visualized using a fluorescence microscope, and cyanine 3 fluorescence was detected after 100% excitation at 568 nm.

### Western blotting

The tissues or cells were lysed with a RIPA buffer (Sigma, USA) containing protease inhibitors (Sigma, USA). Protein quantification was performed using a BCA protein assay kit (Pierce, USA), and 30 μg of total protein were separated in a 12% gel by SDS-polyacrylamide gel electrophoresis (SDS-PAGE) and transferred to a PVDF membrane (Hybond-P, Amersham Biosciences Piscataway, NJ, USA). After being blocked with 5% BSA in Tris-buffered saline with 0.1% Tween 20 (TBST) at room temperature for 2 h, the membrane were probed with primary rabbit anti-C12orf59 antibody (ABGENT, CA) at a dilution of 1:2000 in 5% skim milk powder in TBST at 4°C overnight. After being washed three times with TBST buffer, the blots were incubated with horseradish peroxidase (HRP)-conjugated secondary donkey anti-rabbit at a dilution of 1:10000 for 1–2 h. After washed three times with TBST buffer, the blotting signals were visualized with ECL systems (Pierce, Rockford, IL, USA). β-Actin was used as a loading control and was detected with a rabbit mAb (1:1,000 dilution, Novus).

### Immunohistochemistry

Immunostaining of the paraffin-embedded RCC tissue sections was performed by a similar method to that used in our previous work [36]. The sections were deparaffinized and rehydrated, then boiled for the antigen retrieval. The endogenous hydrogen peroxidase was blocked. After being incubated with 10% BSA, the sections were incubated with anti-C12orf59 antibody (HPA036147, Sigma, USA) used at a 1:300 dilution at 4°C overnight. After being washed in PBS, the sections were treated with a MaxVision HRP-Polymer anti-rabbit IHC Kit (Maixin Bio, Fujian, China) and stained with a DAB kit (Maixin Bio, Fujian, China). The expression of C12orf59 was assessed blindly by two independent investigators. The staining of C12orf59 was scored as the product of the staining intensity (on a scale of 0–3: negative = 0, weak = 1, moderate = 2, strong = 3) and the percentage of cells stained (on a scale of 1–5: 1 = 0%–20%; 2 = 21–40%; 3 = 41–60%; 4 = 61–80%; 5 = 81%–100%), resulting in scores ranging from 0 to 15. We defined two subgroups as follows: the low expression group (scores: 0–5) and the high expression group (scores: 6–15) [37].

### Mutational analyses

The genomic DNA was extracted from the cells or tissue specimens using the DNeasy Tissue Kit (Qiagen). The PCR primers were designed to cover all the exons, intron/exon junctions, proximal promoter and exon 1 region of the full-length NM_153022.2 mRNA transcript (Supplementary Table S1). The PCR product was subjected to Sanger sequencing to determine the presence or absence of mutations. All the mutations were confirmed at least twice, and the sequence tracings were reviewed in the forward and reverse directions by visual inspection.

### Statistical analyses

All the statistical analyses were carried out using the SPSS 18.0 statistical software package. χ^2^ tests were used to compare the expression rates of C12orf59 in ccRCC and their adjacent normal kidney tissues and to determine the associations between the expression of C12orf59, HIF1α, HIF2α, and *VHL* mutation status and the clinico-pathological parameters. The bivariate correlations between the variables were calculated by Spearman's correlation coefficients. The multivariate analysis of the relative effect on survival of each parameter included in the univariate analysis was estimated using the Cox proportional hazards regression model. The overall survival rates were determined according to the Kaplan–Meier method and compared using the log-rank test. *P* < 0.05 was statistically significant.

## SUPPLEMENTARY MATERIALS FIGURES AND TABLES


