# Supplementary material for: Down-regulation of C12orf59 is associated with a poor prognosis and VHL mutations in renal cell carcinoma
Source: Oncotarget. 2016 Jan 7;7(6):6824–34. doi: 10.18632/oncotarget.6829 (PMC4872751; doi:10.18632/oncotarget.6829)
Supplement: Supplementary file 1 [file oncotarget-07-6824-s001.pdf]

Down-regulation of C12orf59 is associated with a poor prognosis and VHL mutations in renal cell carcinoma

Supplementary Materials

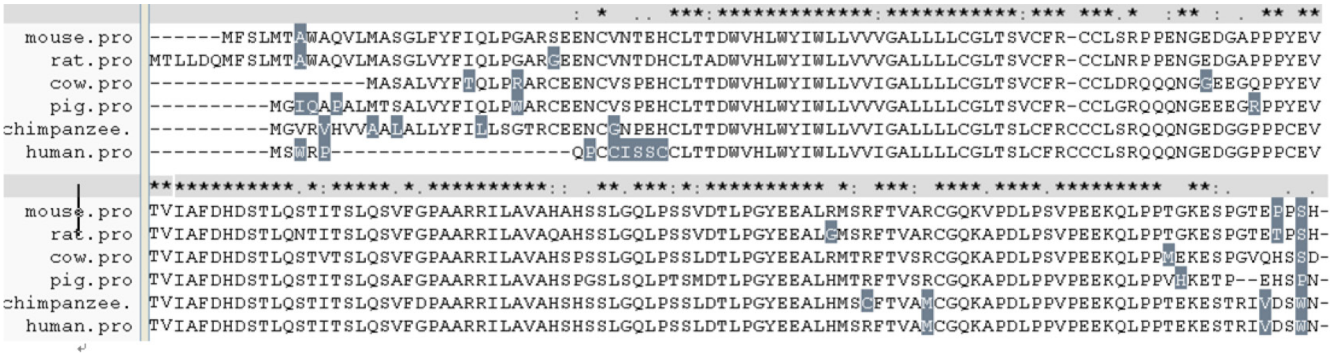

Supplementary Figure S1: Sequence alignment of the C12orf59 proteins from humans, chimpanzees, cows, pigs, mice and rats. The alignment was initially performed with ClustalW and then manually modified. The conserved (100%) residues are labeled with a star above the residues.

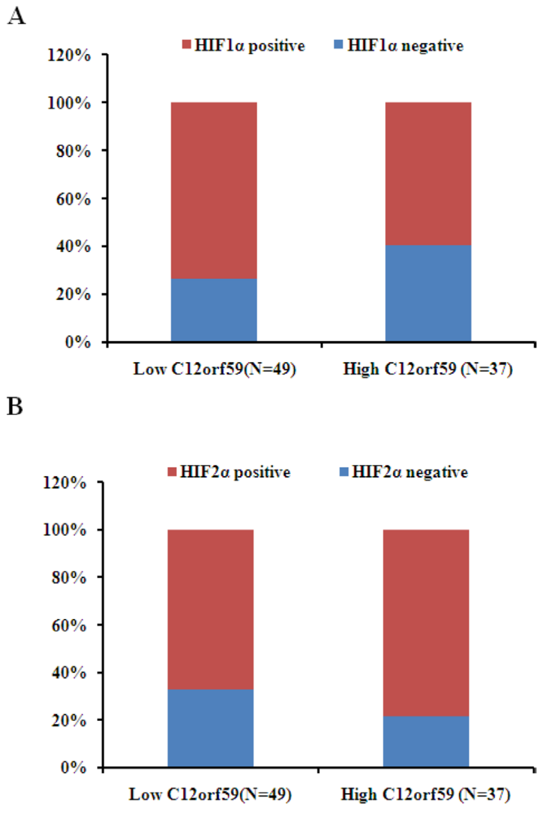

Supplementary Figure S2: Association of C12orf59 expression with HIF1α (A) and HIF2α (B) in 86 ccRCC samples.

Supplementary Table S1: PCR primers for exon amplification

Target	Primer Sequences (5′–3′)	Product Size	Tm
Exon 1	GAAGGCAAAAGGAAGTAAATGTGG	564 bp	61.6
	TGCTCGTTTCCATGCTCACTCTA		62.5
Exon 2	CATTTGGCAAAAGATTCAACTAGC	412 bp	60.6
	ACAGACTCCGAGGGAAGGACA		60.8
Exon 3	CAGCCTGGGCAACAGAGCAA	661 bp	64.3
	GAGCCAGACAGACCCTTGAGAA		60.8
Exon 4	CCTTCTAGCAGAGCAGGAAGCTG	587 bp	62.8
	CCTGTGAACGAAGAGTAAGAACCA		60.5

Supplementary Table S2: Correlation of C12orf59 expression with the clinic-pathologic features of patients with clear cell renal cell carcinoma

Clinico-pathologic variable	Correlation Coefficient	Sig. (2-tailed)	Bias	Std. Error	95% CI
Gender (male/female)	–0.11	0.12	–0.003	0.069	–0.24–0.03
Age (>/< 50 years)	–0.10	0.14	–0.001	0.069	–0.24–0.03
Size (>/< 7 cm)	0.06	0.35	–0.004	0.071	–0.08–0.20
Primary tumor stage (T1–2/T3–4)	–0.17	0.01*	0.001	0.067	–0.23–0.04
Lymph node status (negative/positive)	–0.14	0.04*	0.004	0.061	–0.25–0.01
Distant Metastasis (negative/positive)	–0.17	0.02*	0.002	0.054	–0.26–0.05

Spearman's rank correlation test.

Supplementary Table S3: The list of chromatin remodeling genes with somatic mutations in the 86 ccRCCs

No.	Gene Symbol	Mutation Types
1	ATM	Frame-shift_indel
2	BAP1	Frame-shift_indel,missense,nonsense
3	CREBBP	missense
4	HUWE1	missense
5	IKBKAP	missense
6	JARID1C	Frame-shift_indel,missense,nonsense
7	MYB	missense
8	NCOA2	missense
9	PBRM1	Frame-shift_indel,missense,nonsense
10	RB1	missense
11	SETD2	Frame-shift_indel,splicesite
12	SETDB2	missense
13	SOX9	indel
14	TET2	nonsense
15	USP21	missense

Supplementary Table S4: Correlation between C12orf59 expression and UMPP gene and chromatin remodeling gene mutations in patients with clear cell renal cell carcinoma

Gene	No. of Cases	C12orf59 expression		χ^2	*p*
		low	high		
All cases	86	49	37		
VHL with mutation					
Yes	27	20	7	4.693	0.03
No	59	29	30		
VHL with non-sense mutation or frame-shift mutations					
Yes	19	16	3	7.379	0.007
No	67	33	34		
UMPP gene with mutation					
Yes	45	30	15	3.615	0.057
No	41	19	22		
UMPP gene with non-sense mutation or frame-shift mutations					
Yes	23	19	4	8.415	0.004
No	63	30	33		
Chromatin remodeling gene with mutation					
Yes	32	17	15	0.308	0.579
No	54	32	22		
Chromatin remodeling with non-sense mutation or frame-shift mutations					
Yes	27	15	12	0.032	0.857
No	59	34	25		

Supplementary Table S5: The list of genes encoding the ubiquitin-mediated proteolysis pathway with somatic mutations in the 86 ccRCCs

No.	Gene Symbol	Mutation Types
1	BAP1	Frame-shift_indel, missense, nonsense
2	BIRC6	missense
3	BTRC	missense
4	CBL	missense
5	CUL1	missense
6	CUL3	missense
7	CUL7	missense, indel
8	HERC1	missense
9	HERC2	missense
10	HERC3	missense
11	HUWE1	missense
12	ITCH	nonsense
13	MAP3K1	missense
14	MDM2	missense
15	TRAF6	missense
16	TRIP12	nonsense
17	UBA1	missense
18	UBE2Q2	missense
19	UBE3B	missense
20	UBE4B	missense
21	UBR5	Frame-shift_indel
22	VHL	Frame-shift_indel, indel, missense, nonsense
23	WWP2	missense
